# Extent of Primary DNA Damage Measured by the Comet Assay in Health Professionals Exposed to Antineoplastic Drugs: A Systematic Review and Meta-Analysis

**DOI:** 10.3390/ijerph17020523

**Published:** 2020-01-14

**Authors:** Vincenza Gianfredi, Daniele Nucci, Cristina Fatigoni, Tania Salvatori, Milena Villarini, Massimo Moretti

**Affiliations:** 1 Department of Pharmaceutical Sciences (Unit of Public Health), University of Perugia, Via del Giochetto, 06122 Perugia, Italy; cristina.fatigoni@unipg.it (C.F.); tania.salvatori90@gmail.com (T.S.); milena.villarini@unipg.it (M.V.); 2 Post Graduate School of Hygiene and Preventive Medicine, Department of Experimental Medicine, University of Perugia, Piazzale Gambuli, 06122 Perugia, Italy; 3 Digestive Endoscopy Unit, IRCCS Istituto Oncologico Veneto, Via Gattamelata 64, 35128 Padova, Italy; dnucci.prof@gmail.com

**Keywords:** comet assay, single-cell gel-electrophoresis, antineoplastic drugs, occupational exposure, health personnel

## Abstract

Background: Antineoplastic drugs (ANDs) are a broad group of chemicals showing, at the same time, carcinogenic effects. The potential, albeit true, risk of side effects cannot be accepted, especially if resulting from occupational exposure. The aim of this study was to evaluate the association between occupational exposure to ANDs and the extent of primary DNA damage in health professionals. Methods: A systematic review and meta-analysis was conducted according to the Preferred Reporting Items for Systematic Reviews and Meta-Analyses guidelines. PubMed/Medline, Web of Science, and Scopus were used to perform the literature search. The databases were examined in July 2019. Sub-group, moderator, and cumulative analyses were conducted. The trim and fill method was used in the case of potential publication bias. Results: Twenty studies were included in the qualitative analysis, and 19 in quantitative evaluation. The pooled effect size was 1.27 [(95% confidence interval (CI) = 0.66–1.88), *p* = 0.000] based on 1569 subjects. The moderator analysis by duration of exposure showed a positive association between duration of exposure and primary DNA damage. Conclusions: This systematic review clearly shows a significant association between occupational exposure to ANDs and the extent of primary DNA damage in health professionals. Considering these results, health professionals should be warned against this potential occupational risk.

## 1. Introduction

Antineoplastic drugs (ANDs) are a broad group of chemicals, mainly used in chemotherapy; their anti-proliferative effect was accidentally discovered during the First and Second World Wars, when bone marrow and lymph nodes depletion was registered in soldiers exposed to sulfur mustard [1,2]. However, the anticancer applicability of sulfur mustard was discovered in the late 1940s, and it was first used to treat non-Hodgkin’s lymphoma and leukaemia [3,4]. ANDs can be classified in several modalities, such as, for instance, considering their mechanism of action, chemical structure, origins, or phase-specific toxicity; however, regardless of the classification used, ANDs show, along with their anti-proliferative properties, a certain amount of carcinogenic effects. ANDs are considered “possibly (group 2B), probably (group 2A) and carcinogenic to humans” (group 1) by the International Agency of Research on Cancer (IARC, Lyon, France) [5].

The potential risk of side effects, including secondary cancers, is bearable and accepted by the scientific community and by most cancer patients, as ANDs are able to increase survival. However, the risk cannot be tolerated, especially if resulting from occupational exposure. To date, several studies have shown the potential association between occupational exposure to ANDs and side effects on health professionals, both considering genotoxic [6,7] and non-genotoxic effects (i.e., teratogenicity, infertility) [8,9,10]. Genotoxic effects have been mainly evaluated through molecular epidemiology studies, in which biomarkers have been used to assess the effect of exposure to ANDs [11]. Numerous genotoxic biomarkers have been described in the literature, most of which are used to identify early cytogenetic effects—such as, for instance, micronuclei, sister chromatid exchanges, and chromosome aberrations—or reversible DNA damage—such as DNA adducts and primary DNA damage [4]. The latter is considered as a biomarker of effective biological dose at the target level and can be assessed through the comet assay (also known as the single-cell gel-electrophoresis test). This procedure was first introduced by Östling & Johanson in 1984 [12] and subsequently modified by Singh et al. in 1988 [13]. The standard alkaline procedure of the comet assay (i.e., lysis at pH 10, unwinding, and electrophoresis at pH > 13) allows the detection of both single- and double-strand DNA breaks, apurinic/apyrimidinic sites (i.e., alkali labile lesions) that are expressed as frank strand breaks in the DNA under the alkaline conditions of the assay, as well as open sites formed during DNA repair incision [14]. The comet assay is relatively easy to perform, thanks to the low number of eukaryotic cells needed, and it is a high sensitivity test able to detect a break in the DNA strand every 10^10^ Daltons [15]. Considering these important advantages, the comet assay has been recently considered applicable for occupational exposure biomonitoring [16,17,18].

The first aim of this systematic review and meta-analysis was to identify all the published studies assessing the occupational exposure risk to ANDs for health professionals, measured by the comet assay. The second aim was to retrospectively analyze and synthesize the available evidence. The last study aim was to combine the collected data in order to perform a meta-analytic evaluation of the risk of DNA damage comparing health professionals occupationally exposed to ANDs and health professionals not exposed to ANDs.

## 2. Materials and Methods

The Preferred Reporting Items for Systematic Reviews and Meta-Analyses (PRISMA) guidelines were used in order to complete the following systematic review and meta-analysis [19]. The structured computer literature search was performed using the following electronic databases: PubMed/Medline, Web of Science, and Scopus. The search strategy was developed with a combination of pre-determined keywords, using both Medical Subject Headings (MeSH) and text words terms according to the type of database consulted. In order to identify a proper set of keywords, three components were taken into account: health professional exposure, antineoplastic drugs, and comet. The identified keywords were then combined using Boolean operators AND/OR. The search strategy is available upon request. The databases were examined in July 2019.

### 2.1. Inclusion/Exclusion Criteria

Two researchers independently screened titles and abstracts of the retrieved references, in order to assess compliance with inclusion/exclusion criteria. In order to be included in this review, the studies were required to report the results of primary research evaluating ANDs exposure in health care professionals (both women and men), as well as the risk of ANDs exposure measured with comet assay. Furthermore, we only included studies published in English and with full text available. Besides, only human studies, with original data (e.g., reviews, letters to the editor, and commentaries were excluded), focusing strictly on our outcome of interest expressed as mean ± standard deviation (SD), were included. In order to perform a very comprehensive analysis, reducing the possibility of losing studies, no time filter was added. A more detailed explanation of inclusion/exclusion criteria, according to a population, intervention, comparison, outcomes, and study design (PICOS) [20], is reported in Table 1. The PICOS criteria were extended with time and language filters, as suggested by the Cochrane Collaboration [21].

### 2.2. Data Extraction

Full text was downloaded only for eligible articles. Data extraction was conducted only for articles included according to the previously mentioned criteria. Two researchers independently performed data extraction using a pre-defined and pre-piloted spreadsheet elaborated in Microsoft Excel^®^ for Windows. Any potential disagreement was solved through discussion between the two researchers or, if disagreement persisted, by consulting a third researcher. Data extracted from the original studies were both quantitative and qualitative. Qualitative data recorded included the following: name of first author, year of publication, country where the study was conducted, and preventive measures adopted. Moreover, characteristics of the subjects were also recorded (e.g., age, gender, and job task). The quantitative data extracted were as follows: sample size, ward of the participants, duration of the exposure (expressed in years), and outcome (expressed as mean). In the case of missing details, the corresponding author of the original manuscript was contacted by e-mail. A crosscheck of the reference lists was performed in order to identify further potentially related articles. References were managed using the citation manager software EndNote.

### 2.3. Quality Evaluation

Two researchers independently performed the quality evaluation of the included articles. A validated tool comprising nine items assessing several methodological characteristics of primary studies (i.e., number of exposed and controls, matching for multiple risk factors, and number of cells scored per subject) was used for evaluation. For each item, the score could range from 1 to 3, while the total score could range from 9 to 27 points [7].

### 2.4. Statistical Analysis

The effect size (ES) was calculated based on the mean, standard deviation (SD), and sample size provided per each study. It was estimated by Cohen’s d, reported with its 95% confidence interval (CI). Cohen’s d is the preferred method of ES calculation, and it is calculated as a difference between two means, divided by the variability among the sample. In this way, it is possible to compare results expressed with different units of measures [22]. The comparison was performed between subjects exposed to the antineoplastic drugs and subjects without any type of exposure. In order to reduce the heterogeneity, a random-effect model was applied to conduct the meta-analysis. The heterogeneity among the included studies was evaluated through Chi^2^ and I^2^ tests. An I^2^ value below 25% means no heterogeneity, a value ranging between 25% and 50% is considered as low, while values between 50% and 75% are considered as moderate, lastly a value >75% means high heterogeneity. Potential publication bias was evaluated through visual examination of the funnel plot and by the Egger’s regression asymmetry test. The funnel plot graphically represents the distribution of the retrieved studies considering ES and standard error. It is used for visually detecting potential publication bias based on the symmetry of the plot. However, as the visual examination could be subjective, the Egger’s regression asymmetry test was used as well, being able to provide an objective measure for publication bias. For the Egger’s regression asymmetry test, the statistical significance was set at *p* < 0.10 [23]. If publication bias was detected, a trim and fill method was used in order to adjust for publication bias [24]. The trim and fill method is a statistical approach aimed to estimate potential missing studies, causing the asymmetry of the funnel plot. This method assumes that the studies with the most extreme ES have to be suppressed, adjusting the overall effect estimate [25]. To perform the meta-analysis, the software Prometa3 ^®^ (Internovi, Italy) was used.

### 2.5. Sub-Group and Sensitivity Analysis

In order to reduce the heterogeneity, two sensitivity analyses were conducted, considering the following: (i) only studies with higher quality score (QS ≥ 16), and (ii) combining studies with the same unit of measure. The subgroups analyses are commonly performed because the effect of a measurement might vary among subgroups of subjects, defined by individual characteristics. In order to consolidate the validity of the results, several sub-group analyses were developed, taking into consideration the following: gender (male and female separately), protective equipment used (nothings or gloves and masks versus at least gloves, masks, and air-flow cabinet), and work task (excluding studies that considered nurses not exposed to ANDs as controls).

### 2.6. Cumulative Analysis

A cumulative analysis is a sequence of meta-analyses, aiming to estimate the changes of the ES starting with a single study and adding the other studies one at a time. The cumulative analysis accumulates the results from the first to the latest study, and each successive result includes a synthesis of all previous studies. This analysis expresses the potential consistency of the results [26]. We performed the cumulative analysis both chronologically (adding each study according to the year of publication) and considering the sample size (from smallest to biggest).

### 2.7. Moderator Analysis

To explore potential moderators of these observed effects, owing to a third variable, we examined the variation in ES associated with the continent where the studies were conducted (categorized in Europe, America, and Asia), and year of publication, categorized into three groups (the first group consisted of studies until 2004, the second between 2005 and 2010, and the third after 2010 until today). These three groups were chosen considering that main guidelines on ANDs manipulation—aimed at reducing the occupational risk—were published in 2004 [27], in 2006 [28], and in 2009 [29]. Moreover, a meta-regression analysis was conducted in order to examine the impact of the duration of occupational exposure (expressed in years), and exposed group’s mean age (expressed in years) on ES.

## 3. Results

### 3.1. Literature Search

**Figure 1:**
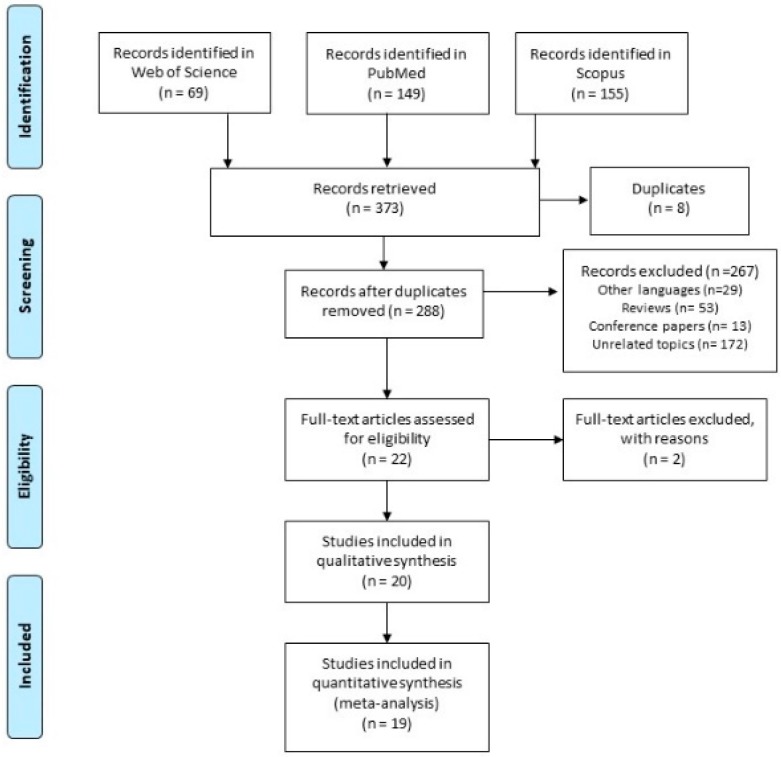
Flow diagram of the selection process.

A total of 373 articles were identified—69 in Web of Science database, 149 in PubMed/Medline, and 155 in Scopus database. A total of 85 documents were immediately excluded because of duplicates. After the preliminary screening by title and abstract, 267 documents were excluded because they were either reviews (*n* = 53), conference papers, or letters to the editor (*n* = 13), or because of unrelated topics (*n* = 172) or different languages (*n* = 29). Out of the 29 articles excluded because of language restriction, we identified articles written in Chinese (*n* = 6), Italian (*n* = 4), French (*n* = 4), Hungarian (*n* = 3), Serbian (*n* =2), Czech (*n* =2), Finnish (*n* =1), German (*n* =1), Japanese (*n* =1), Korean (*n* = 1), Polish (*n* = 1), Portuguese (*n* = 1), Russian (*n* = 1), and Spanish (*n* = 1). Overall, 21 articles were eligible, but one reference was excluded because it was an in vitro study. Figure 1 shows the flow diagram, reporting the selection process. At the end of the screening process, 20 articles were included in the qualitative analysis [30,31,32,33,34,35,36,37,38,39,40,41,42,43,44,45,46,47,48,49], and 19 in the quantitative analysis. One study was removed from the quantitative analysis because, even if the authors had been contacted in order to collect further information, data were not available [49]. Moreover, because four papers reported the results both aggregated and divided by gender, we included the latter data in the sub-group analysis by gender [33,35,40,47]. In order to perform a robust sub-group analysis, when possible, data divided by gender were calculated from the original studies [34]. At the same time, the study conducted by Ursini et al., 2006 [46], showed the results divided by work task, and Kopjar et al., 2001 [37] showed the results divided by protective equipment used; for these reasons, these data were included in the sub-group analysis.

### 3.2. Characteristics of the Included Studies

Most of the included studies were conducted in Western countries, in particular, 10 were conducted in Europe [Italy (*n* = 5), Portugal (*n* = 2), Croatia (*n* = 2), and Austria (*n* = 1)] and 3 in the Americas [United States (*n* = 1) and Brazil (*n* = 2)]. The remaining studies were conducted in Asia, particularly China (*n* = 2), Turkey (*n* = 2), Japan (*n* = 2), and India (*n* = 1). The majority of the studies performed the comet assay using lymphocyte cells, while two studies incorporated the results of the comet assay of buccal cells [31,46]. Most of the studies were published before 2010 (*n* = 17); the oldest study was published in 1999 [45], while the newest retrieved study was published in 2015 [39]. All the included studies except one [49] performed a cross-sectional analysis considering professionals exposed to ANDs compared to others professionals not occupationally exposed to ANDs. Only one study was followed by a follow-up [49]; in this study, the authors estimated the correlation between ANDs’ exposure and comet assay by trend analysis, considering the individual risk with regard to the years of exposure. Concerning the quality evaluation, the score ranged between 11 and 19, with a mean of 16 (Table 2).

### 3.3. Characteristics of the Studied Populations

The sample size of the included studies ranged between 12 and 83 exposed subjects, and 12 and 74 controls. The mean age and SD of the exposed subjects were between 29 ± 5 and 44.17 ± 2.40 years, while for controls, they were 28.23 ± 6.30 and 44.33 ± 1.41 years. The exposed and control groups were perfectly matched for age (*n* = 3), gender (*n* = 9), and smoking (*n* = 6) in several studies. Considering the nutritional intake, a very large majority of the studies either did not match the two groups (*n* = 18), or only partially matched (*n* = 2) by taking into account this confounding variable. In all studies, the considered exposed subjects were nurses, whereas in two studies [34,35], they were the workers producing ANDs. Moreover, pharmacists or pharmacy technicians were also included in the exposed group. Only one study considered physicians as well [38]. Most of the time, the controls comprised workers not occupationally exposed to ANDs. In five studies, the controls were nurses not involved in oncological wards [30,32,40,44,48], while in two studies, no details regarding the characteristics of control subjects were provided [34,35]. Most of the time, exposure to ANDs was assessed through a self-reported questionnaire; environmental monitoring was included in the study design in four studies [39,47,48,49], whereas only three studies [32,42,46] performed biological measurements. Regarding personal protective equipment, gloves and masks were the most frequently used; wearing lab coats, glasses, and caps was also reported. Among the environmental equipment, the use of an air-flow cabinet was reported in only seven studies (Table 2).

Approximately half of the included studies (*n* = 9) scored 100 cells per subject, and eight studies scored a number of cells between 100 and 200; lastly, three studies scored more than 200 cells per subject. The shape of a comet tail represents the entity of DNA damage; the higher the migration of chromosomal DNA from the nucleus, the higher the DNA damage. Several units of measure are used to express the results. In the retrieved studies, tail intensity % was the most used (*n* = 7), followed by tail length (*n* = 6). The DNA damage index was used in three studies; the tail moment, the total comet score, and the log tail length were used once, respectively. Lastly, one study considered a tail factor without any other further explanation [49]. However, all the results were expressed as a mean and SD or standard error of mean (SEM). One study expressed the results as the median [39], and one reported both mean and median [48].

### 3.4. Results of the Meta-Analysis

**Figure 2:**
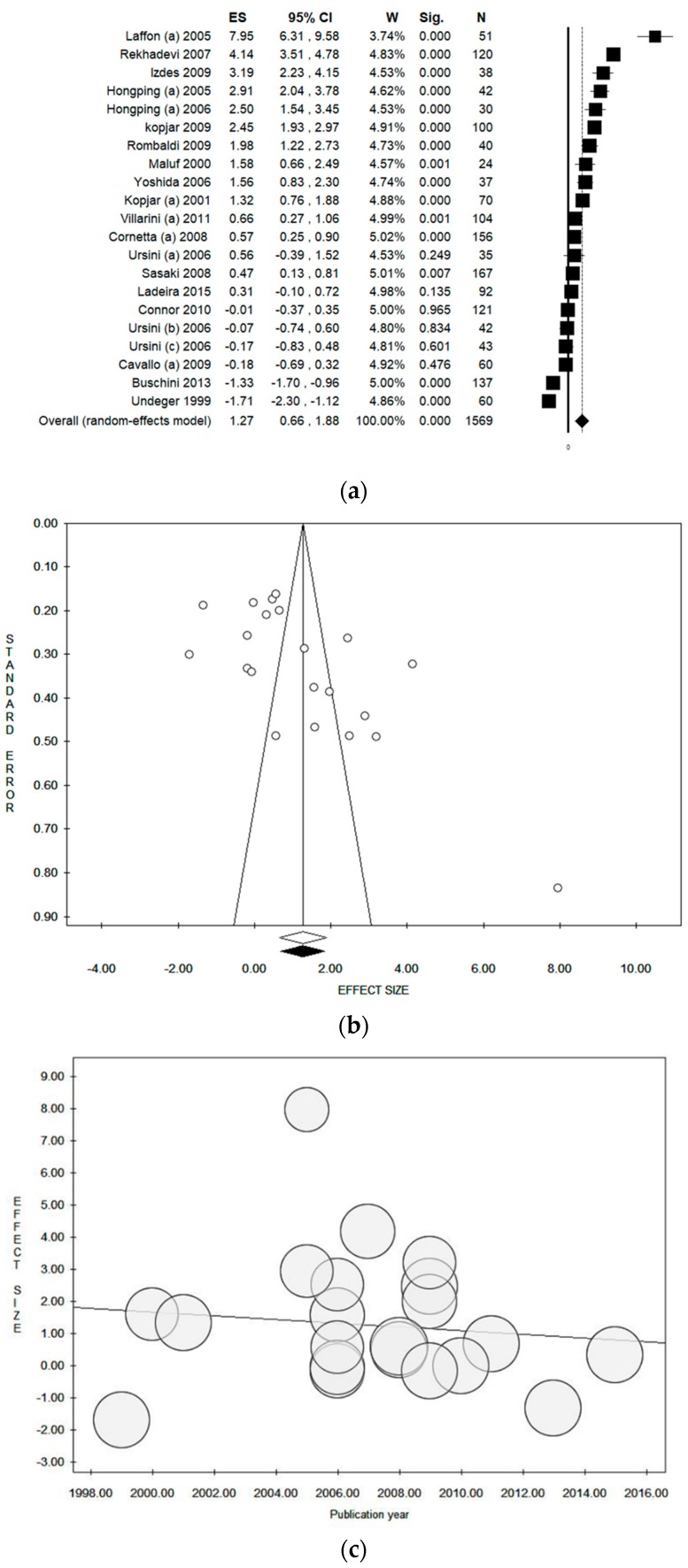
(**a**) Forest plot, (**b**) funnel plot, and (**c**) publication year plot of the meta-analysis assessing the comet assay on lymphocytes cells among professionals occupationally exposed to antineoplastic drugs (ANDs). ES, effect size; CI, confidence interval.

Considering the comet assay performed on peripheral blood lymphocytes, a total of 19 studies were included in the quantitative evaluation. However, one study reported the data separately for pharmacists, nurses working in day hospital, and nurses working in the ward, so they were considered as three independent studies and, for this reason, the meta-analysis included 21 datasets. The pooled ES was 1.27 [(95% CI = 0.66–1.88), *p*-value < 0.001] based on 1569 subjects (Figure 2a), with high statistical heterogeneity (Chi^2^ = 541.17, df = 20, I^2^ = 96.30, *p*-value < 0.001). A potential publication bias was found by the visual assessment of the funnel plot and confirmed by Egger’s linear regression test (intercept 7.56, t = 2.88, *p*-value = 0.010. However, the estimated ES did not change after the trim and fill method was applied (Figure 2b). The publication year plot is depicted in Figure 2c, showing the sequence of publication.

**Figure 3:**
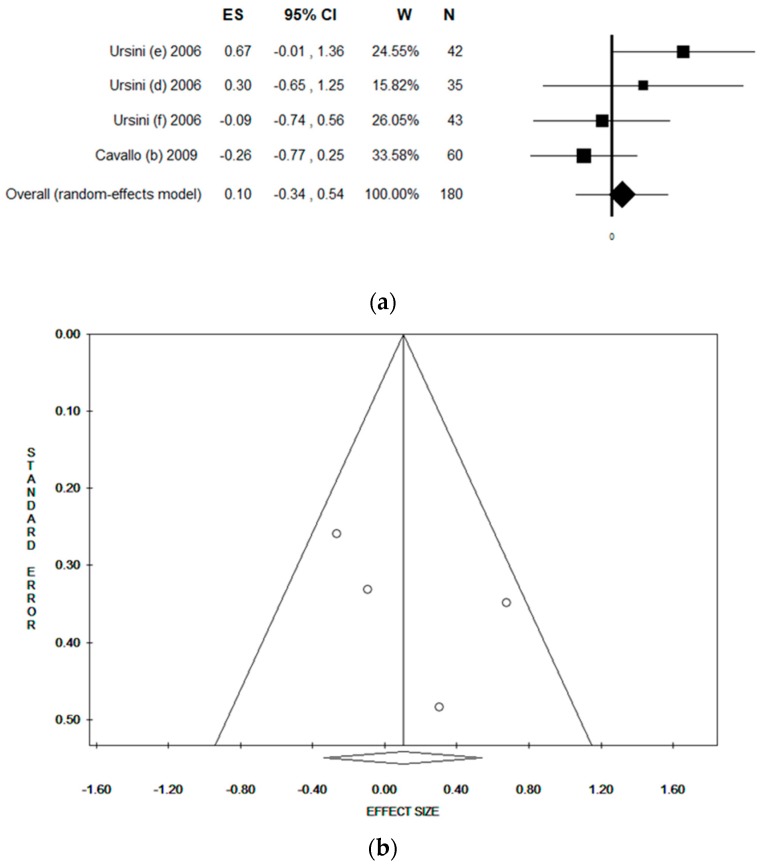
(**a**) Forest plot and (**b**) funnel plot of the meta-analysis assessing the comet assay on buccal cells among professionals occupationally exposed to antineoplastic drugs (ANDs).

Considering the comet assay performed on buccal cells, a total of four datasets were included. The pooled ES was 0.10 [(95% CI = −0.34–0.54), *p*-value = 0.652] based on 180 subjects (Figure 3a). Low statistical heterogeneity was found (Chi^2^ = 5.11, df = 4, I^2^ = 41.27, *p*-value = 0.164). No publication bias was found by the visual assessment of the funnel plot, and confirmed by Egger’s linear regression test (intercept 3.33, t = 1.12, *p*-value = 0.378) (Figure 3b).

### 3.5. Sensitivity Analysis by Quality Score

**Figure 4:**
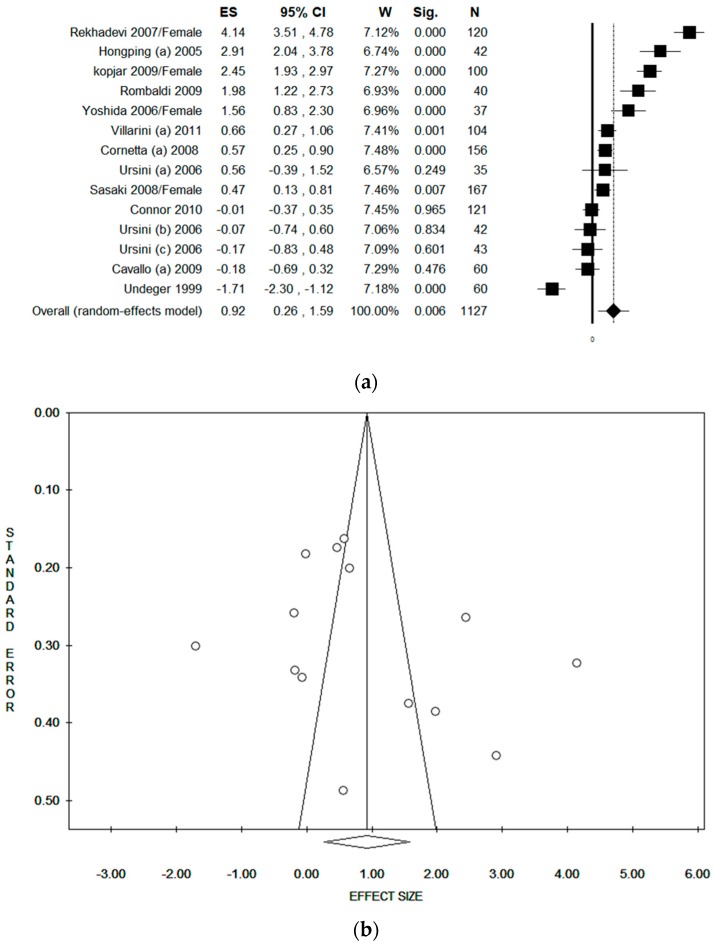
(**a**) Forest plot and (**b**) funnel plot (after trim and fill method) of the meta-analysis assessing the comet assay on lymphocytes cells among professionals occupationally exposed to antineoplastic drugs (ANDs), only including studies with a quality score (QS) ≥16.

In order to reduce heterogeneity, only studies with QS ≥ 16 were included. The pooled ES was 0.92 [(95% CI = 0.26–1.59), *p*-value = 0.006] based on 1127 subjects (Figure 4a). A high statistical heterogeneity was found (Chi^2^ = 302.73, df = 13, I^2^ = 95.71, *p*-value < 0.001). No potential publication bias was found by visual assessment of the funnel plot, and confirmed by Egger’s linear regression test (intercept 4.22, t = 1.08, *p*-value = 0.300) (Figure 4b).

### 3.6. Sub-Group Analysis by Gender

**Figure 5:**
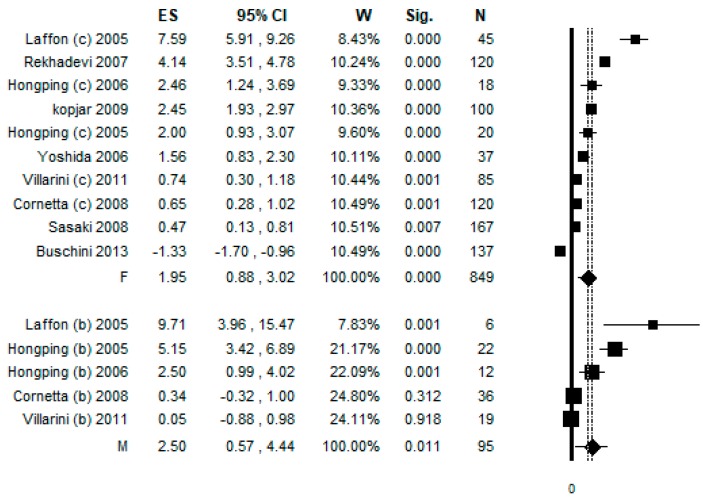
Forest plot of the sub-group analysis by gender assessing the comet assay on lymphocytes cells among professionals occupationally exposed to antineoplastic drugs (ANDs). F, female, M, male.

In order to increase the robustness of the results, a sub-group analysis by gender was performed. The sub-group analysis considering only females included 10 datasets, and the pooled ES was 1.95 [(95% CI = 0.88–3.02), *p*-value < 0.001] based on 849 subjects (Figure 5). High statistical heterogeneity was found (Chi^2^ = 355.84, df = 9, I^2^ = 97.47, *p*-value < 0.001). The sub-group analysis considering only males included five studies and the pooled ES was 2.50 [ (95% CI = 0.57–4.44), *p*-value = 0.011] based on 95 subjects (Figure 5). High statistical heterogeneity was found (Chi^2^ = 42.31, df = 4, I^2^ = 90.55, *p*-value < 0.001). Potential publication bias was found by visual assessment of the funnel plot, and confirmed by Egger’s linear regression test (intercept 7.28, t = 3.15, *p*-value = 0.008). However, after applying the trim and fill method, the estimated ES did not change.

### 3.7. Sub-Group Analysis by Protective Equipment Used

**Figure 6:**
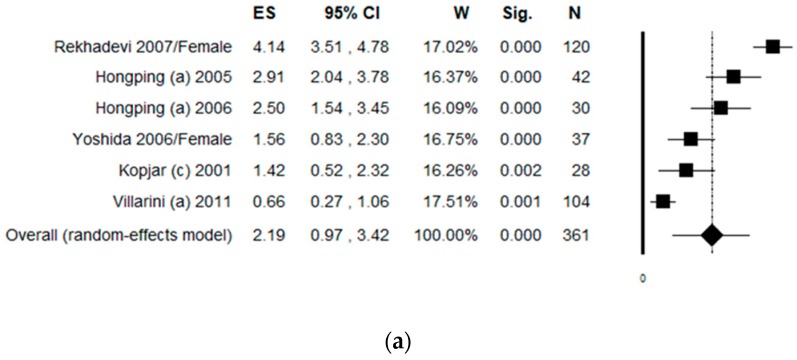

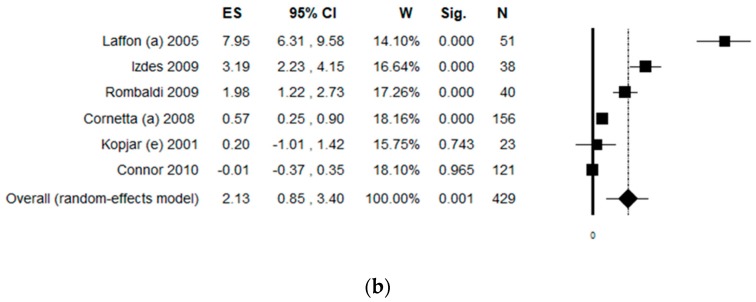
Forest plot of the sub-group analysis by protective equipment used assessing the comet assay on lymphocytes cells among professionals occupationally exposed to antineoplastic drugs (ANDs). (**a**) Without any equipment or at least gloves and masks; (**b**) using at least gloves, masks, and air-flow cabinet.

The sub-group analysis including studies with a low level of protection (considered as no protection at all or use of only gloves and masks) counted six datasets. The pooled ES was 2.19 [(95% CI = 0.97–3.42), *p*-value < 0.001] based on 361 subjects (Figure 6a). High statistical heterogeneity was found (Chi^2^ = 93.63, df = 5, I^2^ = 94.66, *p*-value < 0.001). No publication bias was found by visual assessment of the funnel plot, which was confirmed by Egger’s linear regression test (intercept 6.52, t = 1.30, *p*-value = 0.265). The sub-group analysis considering a high level of protection (at least gloves, masks, and air-flow cabinet), included six studies, and the pooled ES was 2.13 [(95% CI = 0.85–3.40), *p*-value = 0.001] based on 429 subjects (Figure 6b). In this sub-group analysis, Kopjar 2009 was excluded because the exposed group was composed of nurses who used heterogeneous protective equipment (among the 50 subjects, only 3 of them used gloves, masks, and air-flow cabinet, but the results were only shown as aggregated data). High statistical heterogeneity was found (Chi^2^ = 128.11, df = 5, I^2^ = 96.10, *p*-value < 0.001). Potential publication bias was found by visual assessment of the funnel plot, and confirmed by Egger’s linear regression test (intercept 6.97, t = 2.47, *p*-value = 0.069). However, after the trim and fill method was applied, the estimated ES did not change.

### 3.8. Sub-Group Analysis by Work Task

**Figure 7:**
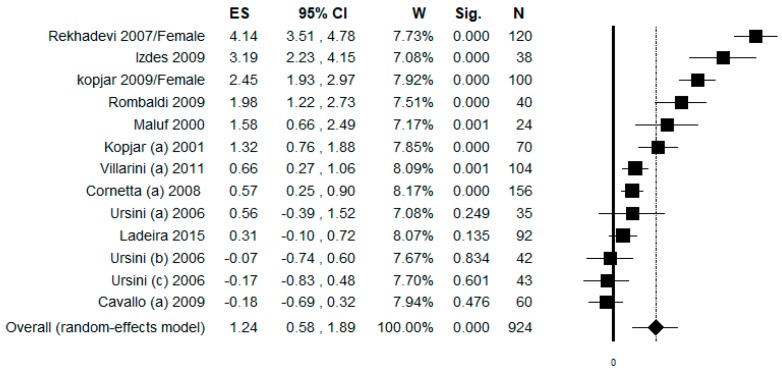
Forest plot of the sub-group analysis by work task (including only studies where controls were other health professionals different from nurses) assessing the comet assay on lymphocytes cells among professionals occupationally exposed to antineoplastic drugs (ANDs).

In the sub-group analysis by work task, we included only studies that considered, as controls, health professionals not exposed to ANDs different from nurses. In this analysis, 13 datasets were included for females and the pooled ES was 1.24 [ (95% CI = 0.58–1.89), *p*-value < 0.001] based on 924 subjects (Figure 7). High statistical heterogeneity was found (Chi^2^ = 217.72, df = 12, I^2^ = 94.49, *p*-value < 0.001). No publication bias was found by visual assessment of the funnel plot and confirmed by Egger’s linear regression test (Intercept 4.68, t = 1.33, *p*-value = 0.209).

### 3.9. Sub-Group Analysis by Continent

**Figure 8:**
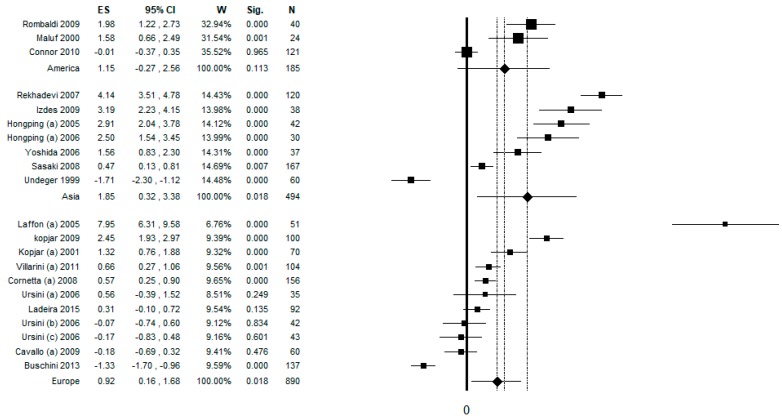
Forest plot of sub-group analysis by continent, assessing the comet assay on lymphocytes cells among professionals occupationally exposed to antineoplastic drugs (ANDs).

In the sub-group analysis by continent, the number of datasets of studies conducted in Europe was 11 and the pooled ES was 0.92 [ (95% CI = 0.16–1.68), *p*-value = 0.018] based on 890 subjects (Figure 8). High statistical heterogeneity was found (Chi^2^= 248.68, df = 10, I^2^ = 95.98, *p*-value < 0.001). The number of datasets of studies conducted in Asia was seven and the pooled ES was 1.85 [(95% CI = 0.32–3.38), *p*-value < 0.001] based on 494 subjects (Figure 8). High statistical heterogeneity was found (Chi^2^ = 262.49, df = 10, I^2^ = 97.42, *p*-value < 0.001). The number of datasets of studies conducted in Americas was three and the pooled ES was 1.15 [ (95% CI = −0.27–2.56), *p*-value < 0.001] based on 185 subjects (Figure 8). High statistical heterogeneity was found (Chi^2^ = 27.64, df = 2, I^2^ = 92.76, *p*-value < 0.001). Potential publication bias was found by visual assessment of the funnel plot and confirmed by Egger’s linear regression test (intercept 8.92, t = 3.54, *p*-value = 0.002). However, after applying the trim and fill method, the estimated ES did not change.

### 3.10. Cumulative Analysis

**Figure 9:**
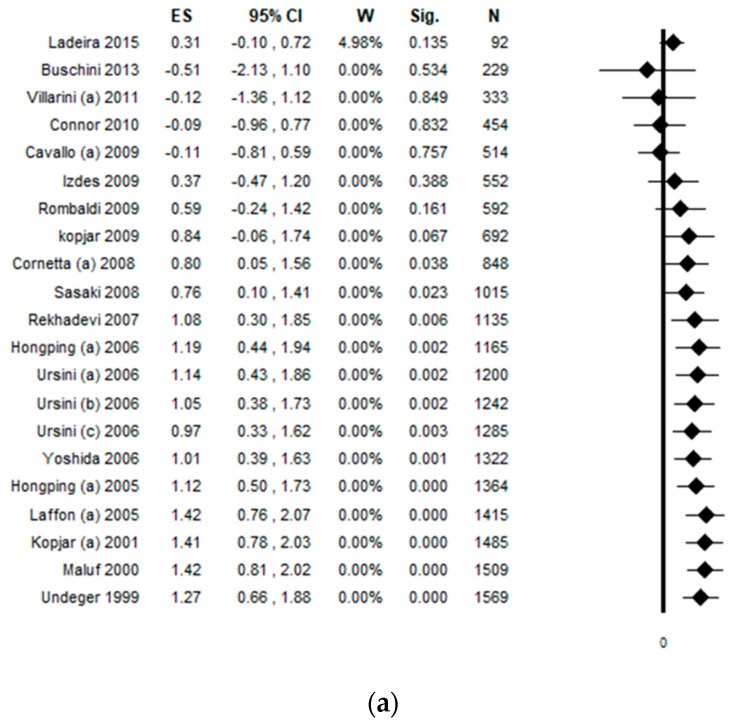

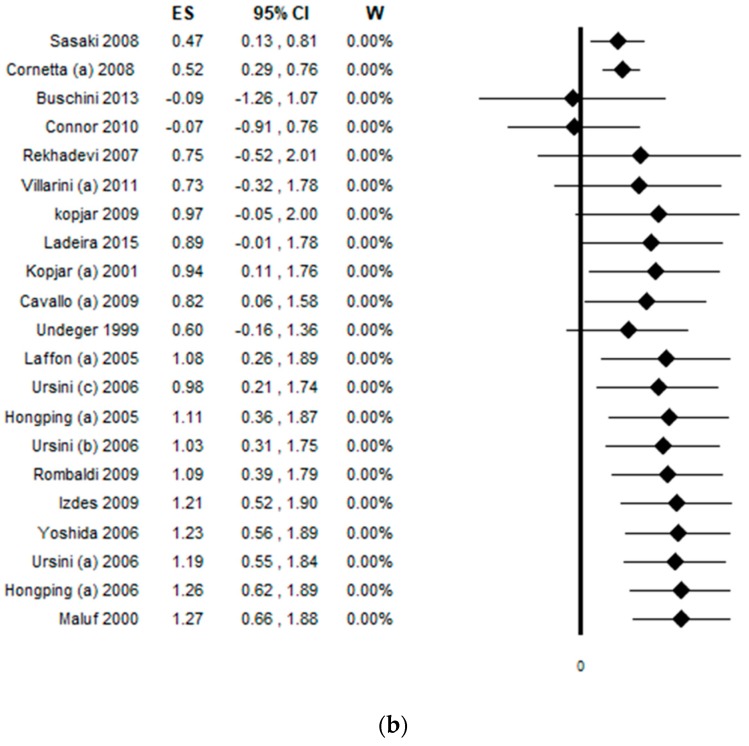
Cumulative analysis by (**a**) year of publication (descending order) and (**b**) effect size (ES) (from the smallest to the biggest) assessing the comet assay on lymphocytes cells among professionals occupationally exposed to antineoplastic drugs (ANDs).

In order to estimate the evolution of the ES across the publications, a cumulative analysis was performed. The cumulative analysis by the year of publication showed a direct association between occupational exposure to ANDs and comet assay, and the results remained stable until the 2008. In 2009, the results started to lose significance, with a large 95% CI (Figure 9a). Referring to the cumulative analysis for sample size, datasets with small sample showed a larger 95% CI. On the contrary, the higher the sample size, the more stable the result (Figure 9b).

### 3.11. Moderator Analysis

**Figure 10:**
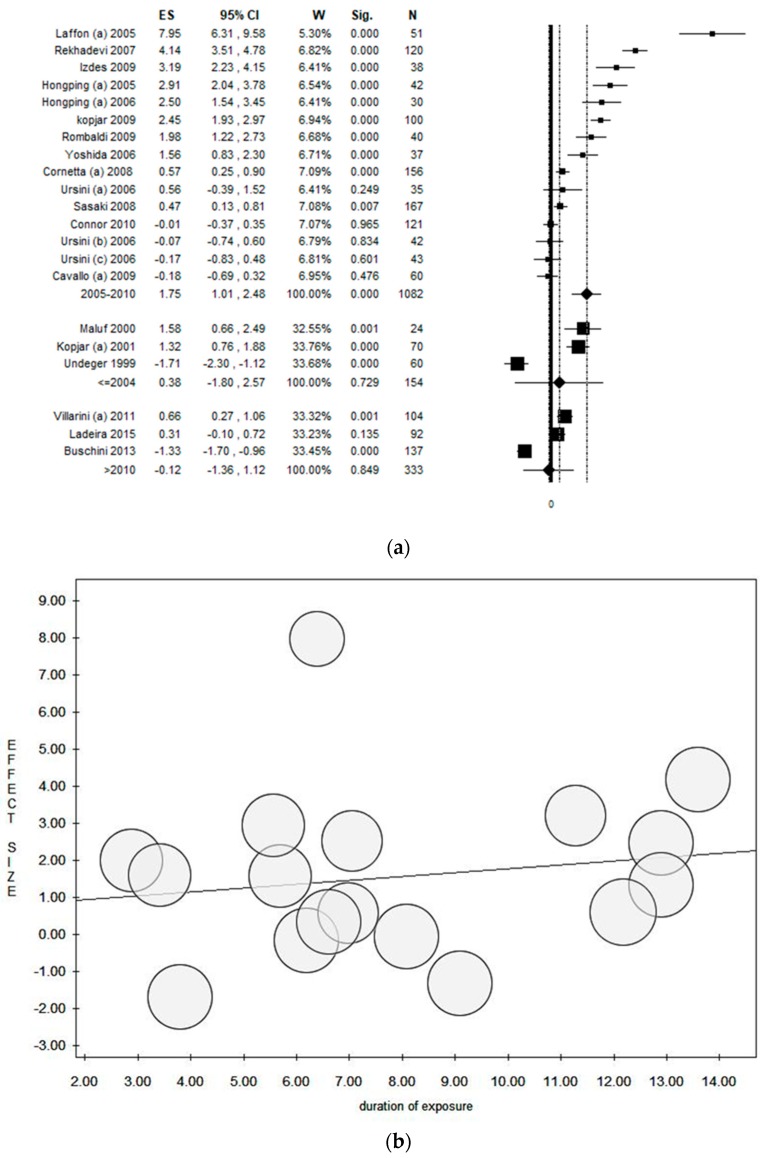

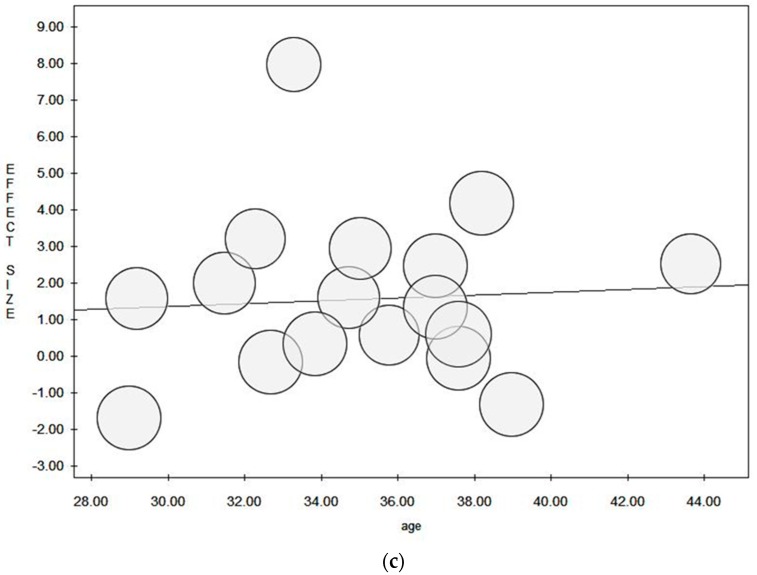
(**a**) Moderator analysis by year of publication (the first group consisted of studies until 2004, the second between 2005 and 2010, and the third after 2010 until today); (**b**) meta-regression analysis by duration of exposure; and (**c**) meta-regression analysis by mean age of exposed groups, assessing the comet assay on lymphocytes cells among professionals occupationally exposed to antineoplastic drugs (ANDs).

Considering the year of publication as covariate in the moderator analysis, the results changed significantly between the three groups of years. Particularly, the early studies (before 2004) did not find a statistically significant association between occupational exposure to ANDs and comet assay. Considering the time period 2005–2010, a higher number of studies were conducted, increasing the sample size (*n* = 1082) and stabilizing the results. However, considering only studies conducted after 2010, only three datasets were included, reducing the sample size (*n* = 333) and increasing the uncertainty of the results, confirmed by the large 95% CI (Figure 10a). The results of the meta-regression analysis by duration of the exposure showed a linear association; a higher duration of exposure was associated with a higher ES (Figure 10b). The association was less clear when the age of exposed groups was considered (Figure 10c).

## 4. Discussion

The current systematic review with meta-analysis—which included 20 studies in the qualitative evaluation and 19 studies in the quantitative analysis—provided data on the association between occupational exposure to ANDs and the extent of primary DNA damage, as evaluated by the comet assay. As some studies separately reported data for gender, work task, and protective equipment, and a total of 21 datasets were considered. The pooled ES based on 1569 subjects indicated a significantly higher extent of primary DNA damage in health professionals exposed to ANDs compared with controls, with an ES of 1.27 [(95% CI = 0.66–1.88), *p*-value < 0.001]. The ES did not change significantly when, in the sub-group analysis, other health professionals different from nurses were considered as controls, even if it was slightly lower [1.24 (95% CI = 0.58–1.89), *p*-value < 0.001].

In order to deeply understand the strength of the association between occupational exposure and primary DNA damage, a sub-group analysis by protective equipment was conducted. When studies that assessed the damage among health professionals who did not use any protective equipment or only masks and gloves were considered, the pooled ES was higher compared with the studies where health professionals used a combination of personal and environmental protective equipment (at least gloves, masks, and airflow cabinet). The importance of protective equipment could explain the differences obtained in the sub-group analysis by continent. Particularly, studies conducted in Asia showed the highest pooled ES compared with studies conducted in the Americas and even more in Europe. As suggested by Hon et al., different standard procedures, protocols, level of education, and legislations across the globe might influence the level of occupational risk, and consequently the conclusions drawn from one country might not be fully applicable to another [50]. Furthermore, in the cumulative analysis by year of publication, the results remained stable until 2008, while from 2009, they started to lose significance, with a large 95% CI. It should be noticed that a large number of guidelines on ANDs’ manipulation, aimed to reduce the occupational risk, were published between 2000 and 2009 [27,28,29,51]. Probably, the reinforcement of protective occupational strategies, the higher attention reserved to the occupational health risk, and particularly the awareness of ANDs dangerousness even at low doses might have played an important role in reducing the occupational exposure risk. However, guidelines alone are not enough, because health professionals need to be empowered with regards to potential and accidental contaminations through tailored training and communication [52]. As a matter of fact, even if health professionals are exposed to a low or very low dose of ANDs, the exposure is nearly daily and prolonged at times. According to our results, the duration of exposure was an important moderator of the association between ANDs exposure and primary DNA damage. This result is in contrast with the data obtained by Mader et al.—the only study that stratified the results according to the duration of exposure (expressed in years) [49]. Considering the sub-group analysis by gender, the pooled ES was marginally higher among men compared with women. However, these data should be read with caution. Firstly, the total datasets assessing primary DNA damage among men were only 5, while 10 datasets performed the comet assay among women; secondly, the men’s sample size was 10 times lower compared with that of the women; thirdly, the 95% CI was wider among men. An important aspect should be considered before the generalization of these results, that is, even though several sub-group analyses were conducted, the value of heterogeneity remained stably high. Indeed, although a sensitivity analysis including only datasets with a high quality score (QS ≥ 16) was conducted, the I^2^ remained around 95%. Only in one case was the heterogeneity below 40%, when datasets performing the comet assay on buccal cells were considered. However, only four datasets provided this data, resulting in a small sample size (*n* = 180 subjects), wide 95% CI, and loss of statistical significance. The high value of heterogeneity found could be explained considering the methodological variation of the comet assay used in the considered primary studies. Indeed, primary papers expressed the results of the comet assay using different types of unit of measures. Moreover, an I^2^ value higher than 90% means that heterogeneity is directly the result of heterogeneity among studies, rather than sampling errors [53]. Even if the pooled ES was estimated by Cohen’s d, allowing comparability, this underlying heterogeneity might have affected the assessment of the I^2^. Another potential explanation of heterogeneity could be the different type of ANDs used or the type of exposure (not only duration or work task, but also doses and therapeutic scheme used). Moreover, even if the primary studies tried to match exposed and control subjects for several confounders, only a few studies perfectly matched for the most important elements, such as smoking, alcohol, and nutritional intake. Nevertheless, there are several strengths in this study: first, this is an extensive systematic review conducted according the PRISMA guidelines, which allowed retrieving a large number of studies; second, the pooled ES was based on 1569 subjects; and third, several sub-group analyses as well as sensitivity and moderator analyses were conducted. Particularly, the ES was estimated in both women and men, considering the work task and the protective equipment used. Moreover, in the meta-regression, the duration of exposure was considered as moderator. Furthermore, whenever we found potential publication bias, a trim and fill method was applied. Even if high heterogeneity was found, a random effect model was considered. Lastly, the pooled ES was estimated both in lymphocytes and buccal cells.

## 5. Conclusions

To conclude, the results of this systematic review with meta-analysis clearly show a statistically significant association between occupational exposure to ANDs and the extent of primary DNA damage. The comet assay method is able to identify reversible DNA damage; in this perspective, the identified damage is still potentially repairable. However, a prolonged exposure, a concomitant action of other risk factors, and the senescence of DNA repair systems might induce a risk of neoplasms or other side effects (such as, for instance, a teratogenic action). Having said that, health professionals should be made aware of this potential occupational risk.

## Figures and Tables

**Table 1:** Detailed inclusion/exclusion criteria, according to a population, intervention, comparison, outcomes, and study design (PICOS) statement extended with a language and time filter.

Search Strategy	Details
Inclusion criteria	P: health professionals occupationally exposed to antineoplastic drugs (ANDs) (female and male)
	I: comet assay measuring primary DNA damage
	C: subjects not exposed to ANDs
	O: mean and standard deviation of primary DNA damage
	S: primary studies (clinical trial, cohort, case-control, cross-sectional)
Exclusion criteria	P: workers not occupationally exposed to ANDs in health care settings
	I: no assessment of genotoxic effects (primary DNA damage) associated to occupational exposure
	O: other outcomes not related to primary DNA damage
	S: not original papers (opinion paper, review article, commentary, letter, article without quantitative data)
Language filter	English
Time filter	No filter (from inception)
Database	PubMed/Medline; Web of Science; Scopus

**Table 2:** Descriptive characteristics of the included studies.

Author, Year	Country	Gender	Mean Age ± SD (Years)	*n* (E/C)‡	Work Task	Mean Exposure ± SD (Years)	Protective Equipment	Cells	Comet Test Mean ± SD	QS/27
Bruschini A et al., 2013	Italy	F	E‡ = 39.0 ± 8.0 C‡ = 40.0 ± 9.0	63/74	Hospital nurses Hospital nurses	9.2 ± 7.2	*n*.a.	lymphocytes	0.95 ± 003 0.99 ± 0.03	15
Cavallo D. et al., 2009 (a)	Italy	M+F	E = 35.2 ± 7.4 C = 34.9 ± 8.5	30/30	Staff hospital ^1^ Administrative employees	*n*.a.	Gloves, caps, overalls, goggles	lymphocytes	10.72 ± 7.04 12.32 ± 10.06	18
Cavallo D. et al., 2009 (b)	Italy	M+F	E = 35.2 ± 7.4 C = 34.9 ± 8.5	30/30	Staff hospital ^1^ Administrative employees	*n*.a.	Gloves, caps, overalls, goggles	buccal	12.00 ± 6.10 14.45 ± 11.7	18
Connor T. et al., 2010	USA	M+F	E = 38.5 ± 10.5 C = 39.9 ± 10.4	68/53	Staff hospital ^2^ involved in oncological wards Staff hospital not involved in oncological wards	*n*.a.	Gloves, gowns, goggles, masks, vertical air-flow cabinet	lymphocytes	53.06 ± 7.32 53.12 ± 7.5	17
Cornetta T. et al., 2008 (a)	Italy	M+F	E = 37.6 ± 6.7 C = 37.0 ± 10.0	83/73	Hospital nurses Administrative employees	12.2 ± 7.3	Gloves, overalls, goggles masks, vertical air-flow cabinet	lymphocytes	1.16 ± 0.82 0.77 ± 0.47	17
Cornetta T. et al., 2008 (b)	Italy	M	*n*.a.	16/20	Hospital nurses Administrative employees	12.2 ± 7.3	Gloves, overalls, goggles masks, vertical air-flow cabinet	lymphocytes	1.13 ± 0.98 0.88 ± 0.45	17
Cornetta T. et al., 2008 (c)	Italy	F	*n*.a.	67/53	Hospital nurses Administrative employees	12.2 ± 7.3	Gloves, overalls, goggles masks, vertical air-flow cabinet	lymphocytes	1.16 ± 0.78 0.73 ± 0.48	17
Hongping D. et al., 2005 (a)	China	M+F	E = 35.0 ± 10.4 C = 36.4 ± 10.0	21/21	Drug technicians *n*.a.	5.6 ± 4.2	Gloves, masks	lymphocytes	1.30 ± 0.29 ° 0.70 ± 0.03 °	16
Hongping D. et al., 2005 (b)	China	M	E = 32.0 ± 10.9 C = 33.0 ± 10.4	11/11	Drug technicians *n*.a.	4.3 ± 3.2	Gloves, masks	lymphocytes	1.35 ± 0.18 ° 0.69 ± 0.02 ^§^	16
Hongping D. et al., 2005 (c)	China	F	E = 38.4 ± 9.2 C = 40.1 ± 8.6	10/10	Drug technicians *n*.a.	7.0 ± 4.8	Gloves, masks	lymphocytes	1.26 ± 0.38 ° 0.72 ± 0.04 ^§^	16
Hongping D. et al., 2006 (a)	China	M+F	E = 43.7 ± 1.1 ^ C = 43.5 ± 1.4 ^	15/15	Drug technicians *n*.a.	7.1 ± 4.35	Gloves, masks	lymphocytes	1.72 ± 0.57 ° 0.71 ± 0.04 °	14
Hongping D. et al., 2006 (b)	China	M	E = 44.2 ± 2.4 ^ C = 42.2 ± 2.9 ^	6/6	Drug technicians *n*.a.	5.8 ± 2.8	Gloves, masks	lymphocytes	1.88 ± 0.66 ^§^ 0.71 ± 0.03 ^§^	14
Hongping D. et al., 2006 (c)	China	F	E = 43.3 ± 1.1 ^ C = 44.3 ± 1.4 ^	9/9	Drug technicians *n*.a.	7.9 ± 5.1	Gloves, masks	lymphocytes	1.62 ± 0.52 ^§^ 0.71 ± 0.05 ^§^	14
Izdes A. et al., 2009	Turkey	M+F	E = 32.3 ± 5.9 C = 33.5 ± 5.1	19/19	Hospital nurses Administrative employees	11.3 ± 4.2	Gloves, masks, vertical air-flow cabinet	lymphocytes	19.89 ± 4.84 6.84 ± 3.16	14
Kopjar N. Garaj-Vrhovac V. 2001 (a)	Croatia	F	E = 37.0 ± 8.9 C = 29.5 ± 8.2	50/20	Hospital nurses Students, administrative employees	12.9 ± 9.4	Gloves, masks, vertical air-flow cabinet	lymphocytes	81.49 ± 4.31 76.01 ± 3.7	12
Kopjar N. Garaj-Vrhovac V. 2001 (b)	Croatia	F	E = 36.5 ± 9.5 C = 29.5 ± 8.2	20/20	Hospital nurses Students, administrative employees	12.1 ± 9.5	Gloves	lymphocytes	83.44 ± 1.49 76.01 ± 3.7	12
Kopjar N. Garaj-Vrhovac V. 2001 (c)	Croatia	F	E = 35.5 ± 9.3 C = 29.5 ± 8.2	8/20	Hospital nurses Students, administrative employees	14.1 ± 8.8	Gloves, masks	lymphocytes	81.6 ± 4.51 76.01 ± 3.7	12
Kopjar N. Garaj-Vrhovac V. 2001 (d)	Croatia	F	E = 37.8 ± 8.4 C = 29.5 ± 8.2	19/20	Hospital nurses Students, administrative employees	13.0 ± 9.9	Gloves, vertical air-flow cabinet	lymphocytes	80.14 ± 5.17 76.01 ± 3.7	12
Kopjar N. Garaj-Vrhovac V. 2001 (e)	Croatia	F	E = 39.3 ± 10.1 C = 29.5 ± 8.2	3/20	Hospital nurses Students, administrative employees	14 ± 12.5	Gloves, masks, vertical air-flow cabinet	lymphocytes	76.8 ± 5.9 76.01 ± 3.7	12
Kopjar N. et al., 2009	Croatia	F	E = 37.0 ± 8.9 C = 37.9 ± 8.9	50/50	Staff hospital ^3^ Students, administrative employees	12.9 ± 9.4	Gloves, masks, vertical air-flow cabinet	lymphocytes	17.46 ± 1.99 14.00 ± 0.14 °	17
Ladeira C. et al., 2015	Portugal	M+F	E = 33.8 ± 1.2 ^ C = 39.3 ± 1.4 ^	46/46	Staff hospital ^4^ Teachers, administrative employees	6.6 ± 0.9 *	*n*.a.	lymphocytes	13.36 * 11.12 *	15
Laffon B. et al., 2005 (a)	Portugal	M+F	E = 33,3 ± 9,2 C = 44,1 ± 8,2	29/22	Hospital nurses Hospital nurses	6.4 ± 6.2	Wearing laboratory coat, mask, gloves vertical air-flow cabinet	lymphocytes	46.46 ± 0.48 ° 42.68 ± 0.47 °	14
Laffon B. et al., 2005 (b)	Portugal	M	*n*.a.	4/2	Hospital nurses Hospital nurses	6.4 ± 6.2	Wearing laboratory coat, mask, gloves vertical air-flow cabinet	lymphocytes	46.65 ± 0.40 ° 42.74 ± 0.41 °	14
Laffon B. et al., 2005 (c)	Portugal	F	*n*.a.	25/20	Hospital nurses Hospital nurses	6.4 ± 6.2	Wearing laboratory coat, mask, gloves vertical air-flow cabinet	lymphocytes	46.43 ± 0.50 ° 42.67 ± 0.49 °	14
Maluf S.W. Anda Erdtmann B. 2000	Brazil	M+F	E = 34.7 ± 5.4 C = 34.4 ± 4.5	12/12	Staff hospital ^5^ *n*.a.	3.4 ± 2.0	*n*.a.	lymphocytes	20.83 ± 10.19 8.08 ± 5.16	15
Rekhadevi P.V. et al., 2007	India	F	E = 38.2 ± 5.6 C = 37.9 ± 5.6	60/60	Hospital nurses General population	13.6 ± 4.8	*n*.a.	lymphocytes	13.66 ± 2.37 6.21 ± 0.92	19
Rombaldi F. et al., 2009	Brazil	M+F	E = 31.5 ± 9.3 C = 28.2 ± 6.3	20/20	Staff hospital ^5^ General population	2.9 ± 3.0	Wearing laboratory coat, mask, gloves vertical air-flow cabinet	lymphocytes	18.86 ± 8.62 6.21 ± 2.78	18
Sasaki M. et al., 2008	Japan	F	E = 37.0 ± 10.0 C = 36.0 ± 9.0	121/46	Hospital nurses Clerks hospital	*n*.a.	*n*.a.	lymphocytes	0.764 ± 0.121 0.711 ± 0.089	16
Undeger U. et al., 1999	Turkey	M+F	E = 29.0 ± 5.0 C = 29.0 ± 5.0	30/30	Hospital nurses Staff hospital (secretaries, nurses techinicians)	3.8 ± 3.1	Gloves, masks, gowns, eye glasses caps	lymphocytes	105.05 ± 36.0 153.8 ± 18.3	19
Ursini C.L. et al., 2006 (a)	Italy	M+F	E = 35.8 ± 9.9 C = 34.9 ± 8.5	5/30	Staff hospital ^5^ Hospital employees	7.0 ± 2.0	Gloves, caps, overalls, goggles	lymphocytes	20.8 ± 10.1 16.1 ± 8.1	18
Ursini C.L. et al., 2006 (b)	Italy	M+F	E = 37.6 ± 5.5 C = 34.9 ± 8.5	12/30	Staff hospital ^5^ Hospital employees	8.1 ± 6.0	Gloves, caps, overalls, goggles	lymphocytes	15.5 ± 9.0 16.1 ± 8.1	18
Ursini C.L. et al., 2006 (c)	Italy	M+F	E = 32.7 ± 7.7 C = 34.9 ± 8.5	13/30	Staff hospital ^5^ Hospital employees	6.2 ± 2.9	Gloves, caps, overalls, goggles	lymphocytes	14.7 ± 7.9 16.1 ± 8.1	18
Ursini C.L. et al., 2006 (d)	Italy	M+F	E = 35.8 ± 9.9 C = 34.9 ± 8.5	5/30	Staff hospital ^5^ Hospital employees	7.0 ± 2.0	Gloves, caps, overalls, goggles	buccal	32.6 ± 18.2 28.6 ± 12.4	18
Ursini C.L. et al., 2006 (d)	Italy	M+F	E = 37.6 ± 5.5 C = 34.9 ± 8.5	12/30	Staff hospital ^5^ Hospital employees	8.1 ± 6.0	Gloves, caps, overalls, goggles	buccal	43.2 ± 36.0 28.6 ± 12.4	18
Ursini C.L. et al., 2006 (e)	Italy	M+F	E = 32.7 ± 7.7 C = 34.9 ± 8.5	13/30	Staff hospital ^5^ Hospital employees	6.2 ± 2.9	Gloves, caps, overalls, goggles	buccal	27.4 ± 13.9 28.6 ± 12.4	18
Villarini M. et al., 2011 (a)	Italy	M+F	E = 39.3 ± 9.6 C = 36.2 ± 11.2	52/52	Staff hospital ^5^ Hospital employees	*n*.a.	Gloves, masks	lymphocytes	2.73 ± 2.02 ° 1.67 ± 1.01 °	18
Villarini M. et al., 2011 (b)	Italy	M	*n*.a.	7/12	Staff hospital ^5^ Hospital employees	*n*.a.	Gloves, masks	lymphocytes	1.82 ± 0.74 ° 1.76 ± 1.42 °	18
Villarini M. et al., 2011 (c)	Italy	F	*n*.a.	45/40	Staff hospital ^5^ Hospital employees	*n*.a.	Gloves, masks	lymphocytes	2.86 ± 2.08 ° 1.64 ± 0.95 °	18
Yoshida J. et al., 2006	Japan	F	E = 29.2 C = 31.6	19/18	Hospital nurses Hospital nurses	5.7	Gloves, masks	lymphocytes	8.80 ± 2.27 6.60 ± 1.07	16

‡ E= exposed, C= controls, ^ ± SE; * median; ° SD estimated from SEM; ^§^ mean and SD estimated from SEM; ^1^ staff hospital (nurses, drug technicians); ^2^ staff hospital (nurses, pharmacists, drug technicians, nursing assistants); ^3^ staff hospital (nurses, medical); ^4^ staff hospital (nurses, pharmacists, drug technicians); ^5^ staff hospital (nurses, pharmacists); M, male; F, female; QS, quality score.
